# The Enlightened Brain: Novel Imaging Methods Focus on Epileptic Networks at Multiple Scales

**DOI:** 10.3389/fncel.2018.00082

**Published:** 2018-03-26

**Authors:** L. Federico Rossi, Dimitri M. Kullmann, Robert C. Wykes

**Affiliations:** ^1^UCL Institute of Ophthalmology, University College London, London, United Kingdom; ^2^Department of Clinical and Experimental Epilepsy, Institute of Neurology, University College London, London, United Kingdom

**Keywords:** epilepsy, wide-field Ca^2+^ imaging, 2-photon imaging, seizure, *in vivo* imaging

## Abstract

Epilepsy research is rapidly adopting novel fluorescence optical imaging methods to tackle unresolved questions on the cellular and circuit mechanisms of seizure generation and evolution. State of the art two-photon microscopy and wide-field fluorescence imaging can record the activity in epileptic networks at multiple scales, from neuronal microcircuits to brain-wide networks. These approaches exploit transgenic and viral technologies to target genetically encoded calcium and voltage sensitive indicators to subclasses of neurons, and achieve genetic specificity, spatial resolution and scalability that can complement electrophysiological recordings from awake animal models of epilepsy. Two-photon microscopy is well suited to study single neuron dynamics during interictal and ictal events, and highlight the differences between the activity of excitatory and inhibitory neuronal classes in the focus and propagation zone. In contrast, wide-field fluorescence imaging provides mesoscopic recordings from the entire cortical surface, necessary to investigate seizure propagation pathways, and how the unfolding of epileptic events depends on the topology of brain-wide functional connectivity. Answering these questions will inform pre-clinical studies attempting to suppress seizures with gene therapy, optogenetic or chemogenetic strategies. Dissecting which network nodes outside the seizure onset zone are important for seizure generation, propagation and termination can be used to optimize current and future evaluation methods to identify an optimal surgical strategy.

## Introduction

Unraveling the mechanisms underlying the development of epilepsy (i.e., epileptogenesis) and the generation of seizures (i.e., ictogenesis) requires one to study brain networks at multiple scales. In many patients, seizures arise from a localized focus, where acquired lesions or congenital abnormalities predispose to aberrant recruitment of neuronal activity in the rest of the brain (Wiebe and Jette, 2012). Yet, even in focal epilepsies, none of the circuitry at the ictogenic focus operates in isolation; rather, it is embedded in an intricate network, where local and distal brain areas are connected by short- and long-range axon collaterals. Indeed, epilepsy can be viewed as a system disorder, whereby both local and dispersed regions of abnormality result in distributed pathological networks (Bernhardt et al., 2013).

Not to miss the forest for the trees, we would ideally need to record in exquisite detail neuronal activity both at the seizure focus and in brain-wide networks. Two state-of-the-art optical imaging technologies, two-photon imaging and wide-field fluorescence imaging, can bridge the gap between neuron-level and whole brain seizure mechanisms, and shed light on the local and global mechanisms triggering epileptic networks. We further argue that the combination of imaging, electrophysiology and optogenetics could identify which neuronal classes, circuits and network nodes are causally involved in the progression of ictogenesis: such “choke points” could be targeted by therapeutic strategies (Paz and Huguenard, 2015).

## Imaging Neuronal Activity at Multiple Scales

**Figure 1:**
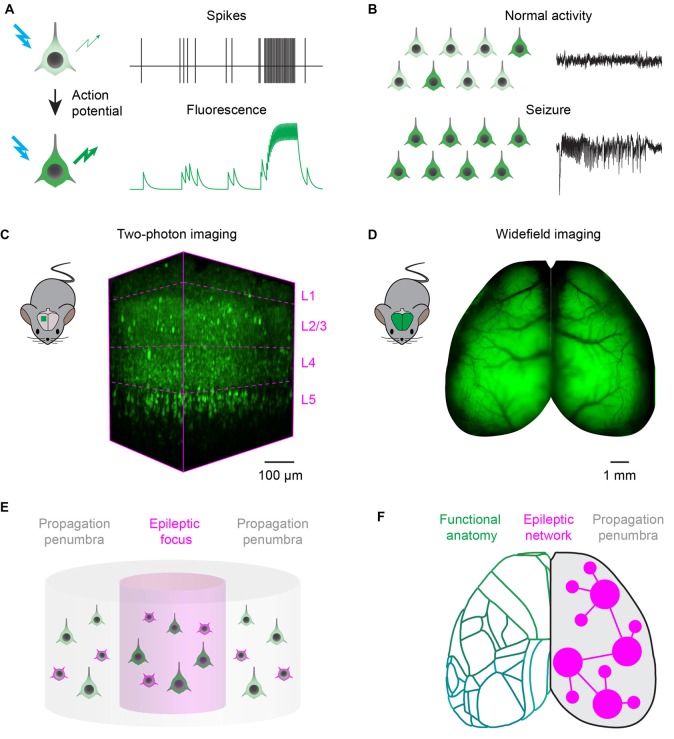
Fluorescence imaging of epileptic activity at multiple scales. **(A)** Fluorescent activity reporters can be targeted to neurons to read out their activity. Reporters change the intensity, or wavelength, of their fluorescence as a function of neuronal activity. **(B)** Fluorescent reporters targeted to neurons at the population level read out the hallmarks of epileptic activity. During normal processing *(top)*, neuronal activity is sparse and EEG is desynchronized. During seizures, activity escalates recruiting most of the neurons in the network and generates hypersynchronous EEG oscillations. **(C)** Two-photon imaging can be used to record neuronal activity with single cell resolution in a small cortical volume. 3D-reconstruction of a 400 × 800 × 650 μm volume recorded *in vivo* in the visual cortex of a transgenic mouse expressing the genetically encoded calcium indicator GCaMP6s in neocortical excitatory neurons across layers *(magenta dotted lines)*. **(D)** Wide-field imaging can be used to record neuronal activity over the entire dorsal cortex of rodents. Example fluorescence image of the dorsal cortex from a mouse expressing the genetically encoded calcium indicator GCaMP6s in all neocortical excitatory neurons (courtesy of Dr. Nicholas A. Steinmetz). **(E)** Two-photon microscopy can record single neuron dynamics during interictal and ictal discharges, and highlight the differences between the activity of excitatory *(green)* and inhibitory *(magenta)* neuronal classes in the focus and propagation penumbra. **(F)** Wide-field fluorescence imaging provides mesoscopic mapping of the functional anatomy of the cortex (*green outlines*, current neuro-anatomical subdivisions of mouse cortex from the Allen Brain Atlas); it can investigate seizure propagation pathways and ask how the unfolding of epileptic events depends on the topology of brain wide functional connectivity.

Imaging technologies based on fluorescent reporters have revolutionized neurophysiology with the ability to read out neuronal activity in unprecedented detail *in vivo*. Fluorescent sensors, when excited at the appropriate wavelength, change their fluorescence intensity, or color, as a function of neuronal activity (Figure 1A). Synthetic organic dyes can be loaded non-invasively into the brain (Tsien, 1981), while protein-based sensors can be genetically encoded (Miyawaki et al., 1997; Nakai et al., 2001). Reporters can be tailored to record a variety of aspects of cellular physiology (Lin and Schnitzer, 2016): voltage sensors can be intercalated in the cellular membrane to read out the voltage across the membrane (Peterka et al., 2011); ion indicators have been developed to measure calcium (Grienberger and Konnerth, 2012), chloride (Arosio and Ratto, 2014), or proton concentrations in the cytosol; and neurotransmitter sensors can detect release at synapses or in the extra-synaptic space (Marvin et al., 2013). Accordingly, the appropriate sensors can report most of the hallmarks of the transition from normal processing to epileptic activity, including abnormal activity of individual neurons (Figure 1A) and the resulting hypersynchronous firing in a neuronal network (Figure 1B).

Optical imaging methods offer the cell-type specificity, spatial resolution and scalability to complement biases and limitations of electrophysiological recordings in epilepsy research. Electrophysiological recordings remain the gold standard to recognize and study epileptic activity: these methods benefit from high temporal resolution and can be applied in human patients. Multi-electrode extracellular probes can record simultaneously from hundreds of neurons (Jun et al., 2017), reaching even to deep structures: yet their readout is spatially restricted and biased to active neurons. Determining cell type firing is difficult, particularly during a seizure when distortions of action potential waveform prevent spike sorting (Merricks et al., 2015). Alternatively, high density surface arrays can be applied to monitor large portions of cortex: however, they pose difficulties to accurately localize neuronal activity due to poor spatial sampling and volume conduction (Viventi et al., 2011).

Specificity is the key: genetically encoded fluorescent reporters can be expressed under the control of cell-type specific promoters (Madisen et al., 2015), recording at once both activity and genetic identity of the target neurons. For instance, excitatory and inhibitory neurons can be tagged with sensors of different colors and recorded simultaneously in different channels with multispectral imaging (Bouchard et al., 2009; Brondi et al., 2012; Dana et al., 2016). Molecular engineering constantly improves and expands the palette of genetically encoded reporters and actuators (Chen et al., 2013; Dana et al., 2016). Transgenic animal generation (Madisen et al., 2015) and designer viral vectors (Chan et al., 2017) are increasingly used to deliver stable non-cytotoxic expression of reporters in neurons. In parallel, cranial window preparations for chronic imaging have been optimized, allowing activity to be monitored for many hours over the course of weeks and, importantly, in awake animals (Goldey et al., 2014). As a result, imaging yields open-ended, unbiased recordings from all the neurons of interest, both active and silent ones.

Two-photon laser scanning microscopy (Denk et al., 1990) can achieve simultaneous recording from many thousands of neurons in a densely packed cortical column (Pachitariu et al., 2016), an impossible target even for the most advanced electrode array. Two-photon excitation is confined to the focal spot by using pulsed infrared lasers, which provide optical sectioning with micrometre-scale spatial resolution. Moreover, infrared light can penetrate deep in brain tissue: the laser focus can be scanned to reconstruct images from all cortical layers (Figure 1C). In order to image larger neuronal circuits simultaneously, 3D scanning optics can be used for volumetric recordings of ~1 mm^3^ cortical columns (Göbel and Helmchen, 2007; Grewe et al., 2011; Nadella et al., 2016). Adaptive optics and three-photon microscopy systems, more robust to optical aberrations arising at increasing imaging depths, achieve recordings in the mouse hippocampus (Ouzounov et al., 2017). Alternatively, cranial windows can be customized with prisms (Andermann et al., 2013) or periscopes (Barretto et al., 2009) to allow coronal imaging of cortical layers and recordings from deep brain structures. Automated pipelines for image registration, segmentation and detection of neuronal activity are also constantly improving and widely available (Pnevmatikakis et al., 2015; Pachitariu et al., 2016).

Wide-field epifluorescence macroscopes (Ratzlaff and Grinvald, 1991) allow one to record activity over the whole cortical surface at fast rates (Mohajerani et al., 2013), representing a substantial advance over electrophysiological recordings performed relatively sparsely at multiple sites (Figure 1D). Wide-field macroscopes are relatively inexpensive to build, with the best optics and cameras that can achieve a spatial resolution of ~50 μm and frame rates up to the kHz range. Wide-field imaging can be used to map the organization of cortical areas and reveal the topology of functional connectivity among them (Kalatsky and Stryker, 2003; Mohajerani et al., 2013). Moreover, wide-field imaging has been combined with multisite electrode probes to correlate cortical activations patterns with neuronal activity recorded in deep brain structures (Xiao et al., 2017).

Recent advances in optics aim to combine the advantages of scanning microscopy and wide-field imaging, towards the tantalizing vision of a single system capable of imaging large fields of view, at fast rates, with single cell resolution. Mesoscopic objectives can be tailored for two-photon imaging over an entire hemisphere of the mouse brain (Ji et al., 2016). In addition, excitation laser pulses can be multiplexed in space or time to scan simultaneously different focal spots and image larger volumes in less time (Cheng et al., 2011; Yang et al., 2016). Finally, holography can create bespoke illumination patterns to achieve scan-less imaging of a selection of neurons of interest (Nikolenko et al., 2008; Bovetti et al., 2017). A more detailed review of the growing number of microscopy techniques available for *in vivo* imaging of neuronal activity has recently been published (Yang and Yuste, 2017).

## Epilepsy in the Spotlight: First Insights From *In Vivo* Imaging Studies

Several recent studies adopted fluorescence imaging methods to investigate the cellular and circuit mechanisms of epileptic networks in animal models. In particular, mice have become a powerful model organism for analyzing genetic diseases, thanks to their genetic tractability. It is possible to derive transgenic lines that harbor mutations in genes found in human forms of genetic epilepsy: for example mouse models of Dravet syndrome (Yu et al., 2006), temporal lobe epilepsy (Chabrol et al., 2010) and migraine associated with seizures (van den Maagdenberg et al., 2004) have been developed. Despite these significant advances, imaging of rare spontaneous seizures have proven difficult, and researches have often resorted to study pharmacologically induced seizures. One of the main challenges ahead will be to develop continuous in-cage imaging of brain activity in freely moving mice prone to spontaneous seizures.

Wide-field calcium imaging was adopted to investigate the spatio-temporal evolution of interictal and ictal discharges and relate it to the functional connectivity underlying normal processing in the awake cortex. Full-blown seizures and brief interictal events were elicited by focal chemoconvulsant application to the primary visual cortex: both types of events started as standing waves, displaying activity that rose and abated quasi-simultaneously in the V1 focus and in connected locations in higher areas (Rossi et al., 2017). Following this common beginning, however, interictal waves decayed to baseline, while seizures persisted and propagated in the cortex. Importantly, this propagation respected the functional connectivity of the visual cortex: areas with a similar retinotopic mapping as the primary focus were recruited before areas that mapped onto different retinal positions (Rossi et al., 2017). These events were so spatially stereotyped that even the prominent oscillations typical of seizures propagated along homotopic connectivity (Rossi et al., 2017).

Once a seizure generalizes, the relationship between patterns of activity and cortical topology becomes more complex: several voltage and calcium imaging studies confirmed that multiple leading regions other than the focus appear to concurrently pace the seizure oscillations (Smith et al., 2016), giving rise to competing spiral waves (Huang et al., 2010; Rossi et al., 2017), colliding planar waves, or more intricate motifs and dynamics (Liou et al., 2017).

In parallel, two-photon calcium imaging has been used to record from excitatory and inhibitory neurons in the ictal penumbra, and elucidate the role of inhibition in restraining dynamics of seizure propagation (Trevelyan et al., 2006, 2007). Similarly to the findings from wide-field studies, neuronal populations within and across cortical layers were recruited in a reliable manner, with stereotyped spatial direction of propagation, supposedly constrained by local connectivity. Interestingly, the temporal dynamics of this recruitment were variable across seizures: such “elasticity” depended on the activity of local GABAergic neurons (Wenzel et al., 2017). Acute breakdown of distant inhibition also allowed the development of a secondary focus, with seizures in this area triggered by input from the original focus (Liou et al., 2017). These results suggest that inhibition not only plays an important role in containing seizure invasion close to adjacent cortex, but also protects areas distant from the seizure focus.

Previous to this work, two-photon imaging was also used to examine which microcircuits participate in inter-ictal discharges in awake animals and how their activity interferes with normal processing. In a chronic model of temporal lobe epilepsy, hippocampal interictal discharges recruited variable cellular dynamics, yet with preferential involvement of GABAergic neurons (Muldoon et al., 2015). In the visual cortex, acutely induced inter-ictal spiking interfered with visual responses evoked in the connected contralateral visual cortex, although this region was not recruited to the epileptiform activity (Petrucco et al., 2017). As inter-ictal spiking is associated with cognitive impairments (Binnie, 2003) and aberrant hippocampal interneuron firing is correlated with cognitive dysfunction (Lewis et al., 2012) these studies suggest a plausible link between inter-ictal spiking, GABAergic activation and cognitive deficits, which could also derive from impaired processing in regions downstream of the ictogenic focus.

Imaging has also played a prominent role in defining the importance of astrocytes in modulating neuronal activity during ictogenesis (Carmignoto and Haydon, 2012). Indeed, while astrocytes are not electrically excitable cells, they signal their activity with prominent intracellular calcium oscillations (Losi et al., 2017). Dysfunctional astrocytes have been proposed to promote ictogenesis modulating neuronal excitability and synchrony (Gómez-Gonzalo et al., 2010; Coulter and Steinhäuser, 2015). Recent recordings *in vivo* called into question these hypotheses, showing that the astrocyte syncytium is indeed recruited into a dramatic “calcium wave” of activation during ictal discharges, but this wave starts after seizure onset and unfolds with spatio-temporal dynamics that seem uncoupled from the underlying neuronal activity (Daniel et al., 2015; Baird-Daniel et al., 2017). These studies suggest that glial activation might not be required for ictal initiation and propagation, as blockade of glial calcium signaling had no impact on seizure dynamics (Baird-Daniel et al., 2017).

## Outlook: Open Questions to Design New Therapeutic Strategies

An ongoing debate regards the dynamics of neuronal firing in the ictogenic network (Szabo et al., 2015). Decades of electrophysiological studies established the classical view of epileptic seizures as hypersynchronous and stereotyped events (McCormick and Contreras, 2001). Synchronicity implies coherent firing in the local population, while stereotypy refers to fixed temporal firing patterns that are repeated across epileptic events. While many recordings from humans and animals confirmed highly coherent firing at the focus and decreased coherence in the regions surrounding the focus (Schevon et al., 2012; Smith et al., 2016), recent analysis of human recordings reported unexpected contrasting results, showing either low coherence but high reproducibility of neuronal spiking patterns (Truccolo et al., 2011) or completely variable recruitment patterns of the same neurons (Bower et al., 2012).

A related question concerns the difference between seizures, and the numerous, brief interictal discharges that occur between them. It is not known if ictal and interictal discharges originate in similar populations, and if the two types of event differ from the very beginning in terms of neuronal recruitment and temporal evolution (Prince and Wilder, 1967; de Curtis and Avanzini, 2001). Understanding these factors would help clarify why one type of event propagates and the other does not (Huberfeld et al., 2011).

Two-photon imaging could provide a unifying description of population dynamics during interictal and ictal discharges, and highlight the differences between activity in the focus and propagation zone (Figure 1E). Current unanswered questions and controversies could be due to the technical pitfalls of electrophysiological recordings, which allow only sparse sampling of the local neuronal population (Wenzel et al., 2017). Two-photon imaging can overcome these limitations, and can be used to ascertain the principles governing population activity in animal models of epilepsy. These principles, in turn, will guide the interpretation of electrophysiological recordings from human subjects: such interpretation is paramount for the correct localization of the ictogenic focus, particularly when brain surgery is the only option available to patients with pharmacoresistant focal epilepsy (Schevon et al., 2012).

Another important set of questions relates to the role of different excitatory or inhibitory neuronal classes, and the conserved circuit motifs connecting them, in generating, propagating and modulating seizure activity (Paz and Huguenard, 2015). A longstanding view states that seizures arise from an imbalance between excitation and inhibition in cortical circuits (Prince and Wilder, 1967), and depend on critical nodes in the circuit (Paz and Huguenard, 2015). Intensely studied examples include feedforward inhibition by parvalbumin positive cells, which have an important role to restrain the propagation of cortical focal seizures (Sessolo et al., 2015), and cortico-thalamo-cortical loops, which underlie spike-wave discharges in absence epilepsies (Sorokin et al., 2016). The combination of two-photon imaging and intersectional genetic strategies can clarify which neuronal classes, and what circuits, are critical for the progression of ictogenesis; such investigations will pave the way for pre-clinical studies attempting to target these “choke points” with gene therapy, optogenetic and chemogenetic strategies (Krook-Magnuson and Soltesz, 2015; Paz and Huguenard, 2015). For instance, inhibition of principal cells with targeted overexpression of K^+^ channels or inhibitory opsins (Wykes et al., 2012), and designer receptors exclusively activated by designer drugs (Kätzel et al., 2014) have been shown to attenuate seizures; alternatively, activation of interneurons with excitatory opsins has also been used to suppress seizures (Krook-Magnuson et al., 2013). While these results are exciting, a recent study suggested that untimely stimulation of parvalbumin positive interneurons might in fact favor the generation of epileptiform discharges (Sessolo et al., 2015; Shiri et al., 2015; Yekhlef et al., 2015). Therefore, a deeper understanding of how these manipulations affect network excitability is needed to avoid off-target effects.

A final question that remains to be elucidated is how the unfolding of epileptic events depends on the topology of brain-wide functional connectivity. In the classical view, seizure spread mimics a brushfire, which progressively wears down inhibitory restraints to recruit contiguous circuitry, as in the “Jacksonian march” seen in the motor cortex. However, somewhat in contrast with this classical view, seizures also spread to distal regions, involving both hemispheres and subcortical centers. The principles that underlie propagation through such a distributed “epileptic network” are incompletely understood: we do not know whether pathological connectivity is required or whether seizures spread along the same functional pathways that support information processing during normal cortical activity. Moreover, once the seizure generalizes it is unclear how different areas interact to maintain the seizure, if pacemaker areas important for seizure maintenance exist, and what pathways promote seizure termination, which is often mysteriously synchronous across the brain (Smith et al., 2016). Answering these questions can explain why surgery might not work in some cases, and, perhaps most crucially, how to optimize current and future evaluation methods to predict an optimal surgical strategy (Goodfellow et al., 2016).

Wide-field calcium imaging could be an important tool to answer these questions, and help devise a predictive modeling framework to quantify the contribution of different network component to ictogenesis (Figure 1F), to improve the outcome of treatment (Goodfellow et al., 2016). The map of the structural and functional connectivity between brain areas can be used as a reference to probe the dynamic interactions of the epileptic focus with other functionally connected regions (Rossi et al., 2017). Multiplexed recordings from excitatory and inhibitory neuronal populations will allow to understand and compare pathways for normal processing and epilepsy, and dissect how the breakdown of inhibition promotes seizure propagation (Trevelyan et al., 2007; Schevon et al., 2012). Finally, imaging during generalized seizures, and analysis of differences and commonalities between various pharmacological and genetic models, can be used to identify nodes outside the focus important for seizure generation, maintenance and termination (Liou et al., 2017). For example, it has been proposed that seizures, analogously to cardiac arrhythmias, could be terminated by the mutual annihilation of spiral waves and planar waves originating from different seizing regions: this would have implications for electrical stimulation or optogenetic strategies to disrupt seizures (Viventi et al., 2011). Lastly, wide field imaging can also reveal the causal interaction between seizures and other circuit dysfunctions such as cortical and subcortical spreading depression (Farkas et al., 2008), which have been implicated in post-ictal headache (Charles and Baca, 2013) and sudden unexpected death in epilepsy (Aiba and Noebels, 2015).

## Author Contributions

LFR, DMK and RCW contributed to writing this Perspective article.

## Conflict of Interest Statement

The authors declare that the research was conducted in the absence of any commercial or financial relationships that could be construed as a potential conflict of interest.

## References

[B1] Aiba I. Noebels J. L. (2015). Spreading depolarization in the brainstem mediates sudden cardiorespiratory arrest in mouse SUDEP models. Sci. Transl. Med. 7:282ra46. 10.1126/scitranslmed.aaa4050 25855492 PMC4852131

[B2] Andermann M. L. Gilfoy N. B. Goldey G. J. Sachdev R. N. S. Wölfel M. McCormick D. A.. (2013). Chronic cellular imaging of entire cortical columns in awake mice using microprisms. Neuron 80, 900–913. 10.1016/j.neuron.2013.07.052 24139817 PMC3840091

[B3] Arosio D. Ratto G. M. (2014). Twenty years of fluorescence imaging of intracellular chloride. Front. Cell. Neurosci. 8:258. 10.3389/fncel.2014.00258 25221475 PMC4148895

[B4] Baird-Daniel E. Daniel A. G. S. Wenzel M. Li D. Liou J. Laffont P.. (2017). Glial calcium waves are triggered by seizure activity and not essential for initiating ictal onset or neurovascular coupling. Cereb. Cortex 27, 3318–3330. 10.1093/cercor/bhx072 28369176 PMC6433182

[B5] Barretto R. P. J. Messerschmidt B. Schnitzer M. J. (2009). *In vivo* fluorescence imaging with high-resolution microlenses. Nat. Methods 6, 511–512. 10.1038/nmeth.1339 19525959 PMC2849805

[B6] Bernhardt B. C. Hong S. Bernasconi A. Bernasconi N. (2013). Imaging structural and functional brain networks in temporal lobe epilepsy. Front. Hum. Neurosci. 7:624. 10.3389/fnhum.2013.00624 24098281 PMC3787804

[B7] Binnie C. D. (2003). Cognitive impairment during epileptiform discharges: is it ever justifiable to treat the EEG? Lancet Neurol. 2, 725–730. 10.1016/s1474-4422(03)00584-2 14636777

[B8] Bouchard M. B. Chen B. R. Burgess S. A. Hillman E. M. C. (2009). Ultra-fast multispectral optical imaging of cortical oxygenation, blood flow, and intracellular calcium dynamics. Opt. Express 17, 15670–15678. 10.1364/OE.17.015670 19724566 PMC2760073

[B9] Bovetti S. Moretti C. Zucca S. Dal Maschio M. Bonifazi P. Fellin T. (2017). Simultaneous high-speed imaging and optogenetic inhibition in the intact mouse brain. Sci. Rep. 7:40041. 10.1038/srep40041 28053310 PMC5215385

[B10] Bower M. R. Stead M. Meyer F. B. Marsh W. R. Worrell G. A. (2012). Spatiotemporal neuronal correlates of seizure generation in focal epilepsy. Epilepsia 53, 807–816. 10.1111/j.1528-1167.2012.03417.x 22352423 PMC3339564

[B11] Brondi M. Sato S. S. Rossi L. F. Ferrara S. Ratto G. M. (2012). Finding a needle in a haystack: identification of EGFP tagged neurons during calcium imaging by means of two-photon spectral separation. Front. Mol. Neurosci. 5:96. 10.3389/fnmol.2012.00096 23112759 PMC3482699

[B12] Carmignoto G. Haydon P. G. (2012). Astrocyte calcium signaling and epilepsy. Glia 60, 1227–1233. 10.1002/glia.22318 22389222 PMC4532388

[B90] Chabrol E. Navarro V. Provenzano G. Cohen I. Dinocourt C. Rivaud-Péchoux S.. (2010). Electroclinical characterization of epileptic seizures in leucine-rich, glioma-inactivated 1-deficient mice. Brain 133, 2749–2762. 10.1093/brain/awq171 20659958 PMC2929330

[B13] Chan K. Y. Jang M. J. Yoo B. B. Greenbaum A. Ravi N. Wu W.. (2017). Engineered adeno-associated viruses for efficient and noninvasive gene delivery throughout the central and peripheral nervous systems. Nat. Neurosci. 20, 1172–1179. 10.1038/nn.4593 28671695 PMC5529245

[B14] Charles A. C. Baca S. M. (2013). Cortical spreading depression and migraine. Nat. Rev. Neurol. 9, 637–644. 10.1038/nrneurol.2013.192 24042483

[B15] Chen T. W. Wardill T. J. Sun Y. Pulver S. R. Renninger S. L. Baohan A.. (2013). Ultrasensitive fluorescent proteins for imaging neuronal activity. Nature 499, 295–300. 10.1038/nature12354 23868258 PMC3777791

[B16] Cheng A. Gonçalves J. T. Golshani P. Arisaka K. Portera-Cailliau C. (2011). Simultaneous two-photon calcium imaging at different depths with spatiotemporal multiplexing. Nat. Methods 8, 139–142. 10.1038/nmeth.1552 21217749 PMC3076599

[B17] Coulter D. A. Steinhäuser C. (2015). Role of astrocytes in epilepsy. Cold Spring Harb. Perspect. Med. 5:a022434. 10.1101/cshperspect.a022434 25732035 PMC4355248

[B18] Dana H. Mohar B. Sun Y. Narayan S. Gordus A. Hasseman J. P.. (2016). Sensitive red protein calcium indicators for imaging neural activity. Elife 5:e12727. 10.7554/eLife.12727 27011354 PMC4846379

[B19] Daniel A. G. S. Laffont P. Zhao M. Ma H. Schwartz T. H. (2015). Optical electrocorticogram (OECoG) using wide-field calcium imaging reveals the divergence of neuronal and glial activity during acute rodent seizures. Epilepsy Behav. 49, 61–65. 10.1016/j.yebeh.2015.04.036 25976183

[B20] de Curtis M. Avanzini G. (2001). Interictal spikes in focal epileptogenesis. Prog. Neurobiol. 63, 541–567. 10.1016/s0301-0082(00)00026-5 11164621

[B21] Denk W. Strickler J. Webb W. (1990). Two-photon laser scanning fluorescence microscopy. Science 248, 73–76. 10.1126/science.2321027 2321027

[B22] Farkas E. Pratt R. Sengpiel F. Obrenovitch T. P. (2008). Direct, live imaging of cortical spreading depression and anoxic depolarisation using a fluorescent, voltage-sensitive dye. J. Cereb. Blood Flow Metab. 28, 251–262. 10.1038/sj.jcbfm.9600569 17971792 PMC2653938

[B23] Göbel W. Helmchen F. (2007). New angles on neuronal dendrites *in vivo*. J. Neurophysiol. 98, 3770–3779. 10.1152/jn.00850.2007 17898141

[B24] Goldey G. J. Roumis D. K. Glickfeld L. L. Kerlin A. M. Reid R. C. Bonin V.. (2014). Removable cranial window strategies for long-term two-photon imaging in awake mice. Nat. Protoc. 9, 2515–2538. 10.1038/nprot.2014.165 25275789 PMC4442707

[B25] Gómez-Gonzalo M. Losi G. Chiavegato A. Zonta M. Cammarota M. Brondi M.. (2010). An excitatory loop with astrocytes contributes to drive neurons to seizure threshold. PLoS Biol. 8:e1000352. 10.1371/journal.pbio.1000352 20405049 PMC2854117

[B26] Goodfellow M. Rummel C. Abela E. Richardson M. P. Schindler K. Terry J. R. (2016). Estimation of brain network ictogenicity predicts outcome from epilepsy surgery. Sci. Rep. 6:29215. 10.1038/srep29215 27384316 PMC4935897

[B27] Grewe B. F. Voigt F. F. van ’t Hoff M. Helmchen F. (2011). Fast two-layer two-photon imaging of neuronal cell populations using an electrically tunable lens. Biomed. Opt. Express 2, 2035–2046. 10.1364/BOE.2.002035 21750778 PMC3130587

[B28] Grienberger C. Konnerth A. (2012). Imaging calcium in neurons. Neuron 73, 862–885. 10.1016/j.neuron.2012.02.011 22405199

[B29] Huang X. Xu W. Liang J. Takagaki K. Gao X. Wu J. Y. (2010). Spiral wave dynamics in neocortex. Neuron 68, 978–990. 10.1016/j.neuron.2010.11.007 21145009 PMC4433058

[B30] Huberfeld G. Menendez de la Prida L. Pallud J. Cohen I. Le Van Quyen M. Adam C.. (2011). Glutamatergic pre-ictal discharges emerge at the transition to seizure in human epilepsy. Nat. Neurosci. 14, 627–634. 10.1038/nn.2790 21460834

[B31] Ji N. Freeman J. Smith S. L. (2016). Technologies for imaging neural activity in large volumes. Nat. Neurosci. 19, 1154–1164. 10.1038/nn.4358 27571194 PMC5244827

[B32] Jun J. J. Steinmetz N. A. Siegle J. H. Denman D. J. Bauza M. Barbarits B.. (2017). Fully integrated silicon probes for high-density recording of neural activity. Nature 551, 232–236. 10.1038/nature24636 29120427 PMC5955206

[B33] Kalatsky V. A. Stryker M. P. (2003). New paradigm for optical imaging: temporally encoded maps of intrinsic signal. Neuron 38, 529–545. 10.1016/S0896-6273(03)00286-1 12765606

[B34] Kätzel D. Nicholson E. Schorge S. Walker M. C. Kullmann D. M. (2014). Chemical-genetic attenuation of focal neocortical seizures. Nat. Commun. 5:3847. 10.1038/ncomms4847 24866701 PMC4050272

[B36] Krook-Magnuson E. Armstrong C. Oijala M. Soltesz I. (2013). On-demand optogenetic control of spontaneous seizures in temporal lobe epilepsy. Nat. Commun. 4:1376. 10.1038/ncomms2376 23340416 PMC3562457

[B35] Krook-Magnuson E. Soltesz I. (2015). Beyond the hammer and the scalpel: selective circuit control for the epilepsies. Nat. Neurosci. 18, 331–338. 10.1038/nn.3943 25710834 PMC4340083

[B37] Lewis D. A. Curley A. A. Glausier J. R. Volk D. W. (2012). Cortical parvalbumin interneurons and cognitive dysfunction in schizophrenia. Trends Neurosci. 35, 57–67. 10.1016/j.tins.2011.10.004 22154068 PMC3253230

[B38] Lin M. Z. Schnitzer M. J. (2016). Genetically encoded indicators of neuronal activity. Nat. Neurosci. 19, 1142–1153. 10.1038/nn.4359 27571193 PMC5557009

[B39] Liou A. J. Ma H. Wenzel M. Zhao M. Baird-daniel E. Schevon C. A. (2017). Role of inhibitory control in modulating spread of focal ictal activity. bioRxiv10.1093/brain/awy116 PMC6022627 29757347

[B40] Losi G. Mariotti L. Sessolo M. Carmignoto G. (2017). New tools to study astrocyte Ca^2+^ signal dynamics in brain networks *in vivo*. Front. Cell. Neurosci. 11:134. 10.3389/fncel.2017.00134 28536505 PMC5422467

[B41] Madisen L. Garner A. R. Shimaoka D. Chuong A. S. Klapoetke N. C. Li L.. (2015). Transgenic mice for intersectional targeting of neural sensors and effectors with high specificity and performance. Neuron 85, 942–958. 10.1016/j.neuron.2015.02.022 25741722 PMC4365051

[B42] Marvin J. S. Borghuis B. G. Tian L. Cichon J. Harnett M. T. Akerboom J.. (2013). An optimized fluorescent probe for visualizing glutamate neurotransmission. Nat. Methods 10, 162–170. 10.1038/nmeth.2333 23314171 PMC4469972

[B43] McCormick D. A. Contreras D. (2001). On the cellular and network bases of epileptic seizures. Ann. Rev. Physiol. 63, 815–846. 10.1146/annurev.physiol.63.1.815 11181977

[B44] Merricks E. M. Smith E. H. McKhann G. M. Goodman R. R. Bateman L. M. Emerson R. G.. (2015). Single unit action potentials in humans and the effect of seizure activity. Brain 138, 2891–2906. 10.1093/brain/awv208 26187332 PMC4671476

[B45] Miyawaki A. Llopis J. Heim R. McCaffery J. M. Adams J. A. Ikura M.. (1997). Fluorescent indicators for Ca^2+^ based on green fluorescent proteins and calmodulin. Nature 388, 882–887. 10.1038/42264 9278050

[B46] Mohajerani M. H. Chan A. W. Mohsenvand M. LeDue J. Liu R. Mcvea D. A.. (2013). Spontaneous cortical activity alternates between motifs defined by regional axonal projections. Nat. Neurosci. 16, 1426–1435. 10.1038/nn.3499 23974708 PMC3928052

[B47] Muldoon S. F. Villette V. Tressard T. Malvache A. Reichinnek S. Bartolomei F.. (2015). GABAergic inhibition shapes interictal dynamics in awake epileptic mice. Brain 138, 2875–2890. 10.1093/brain/awv227 26280596

[B48] Nadella K. M. N. S. Roš H. Baragli C. Griffiths V. A. Konstantinou G. Koimtzis T.. (2016). Random-access scanning microscopy for 3D imaging in awake behaving animals. Nat. Methods 13, 1001–1004. 10.1038/nmeth.4033 27749836 PMC5769813

[B49] Nakai J. Ohkura M. Imoto K. (2001). A high signal-to-noise Ca^2+^ probe composed of a single green fluorescent protein. Nat. Biotechnol. 19, 137–141. 10.1038/84397 11175727

[B50] Nikolenko V. Watson B. O. Araya R. Woodruff A. Peterka D. S. Yuste R. (2008). SLM microscopy: scanless two-photon imaging and photostimulation using spatial light modulators. Front. Neural Circuits 2:5. 10.3389/neuro.04.005.2008 19129923 PMC2614319

[B51] Ouzounov D. G. Wang T. Wang M. Feng D. D. Horton N. G. Cruz-Hernández J. C.. (2017). *In vivo* three-photon imaging of activity of GCaMP6-labeled neurons deep in intact mouse brain. Nat. Methods 14, 388–390. 10.1038/nmeth.4183 28218900 PMC6441362

[B52] Pachitariu M. Stringer C. Schröder S. Dipoppa M. Rossi L. F. Carandini M. (2016). Suite2p: beyond 10,000 neurons with standard two-photon microscopy. bioRxiv 61507.

[B53] Paz J. T. Huguenard J. R. (2015). Microcircuits and their interactions in epilepsy: is the focus out of focus? Nat. Neurosci. 18, 351–359. 10.1038/nn.3950 25710837 PMC4561622

[B54] Peterka D. S. Takahashi H. Yuste R. (2011). Imaging voltage in neurons. Neuron 69, 9–21. 10.1016/j.neuron.2010.12.010 21220095 PMC3387979

[B55] Petrucco L. Pracucci E. Brondi M. Ratto G. M. Landi S. (2017). Epileptiform activity in the mouse visual cortex interferes with cortical processing in connected areas. Sci. Rep. 7:40054. 10.1038/srep43808 28071688 PMC5223162

[B56] Pnevmatikakis E. A. Soudry D. Gao Y. Machado T. A. Merel J. Pfau D.. (2015). Simultaneous denoising, deconvolution, and demixing of calcium imaging data. Neuron 89, 285–299. 10.1016/j.neuron.2015.11.037 26774160 PMC4881387

[B57] Prince D. A. Wilder B. J. (1967). Control mechanisms of cortical epileptogenic foci. “Surround” inhibition. Arch Neurol. 16, 194–202. 10.1001/archneur.1967.00470200082007 6018049

[B58] Ratzlaff E. H. Grinvald A. (1991). A tandem-lens epifluorescence macroscope: hundred-fold brightness advantage for wide-field imaging. J. Neurosci. Methods 36, 127–137. 10.1016/0165-0270(91)90038-2 1905769

[B59] Rossi L. F. Wykes R. C. Kullmann D. M. Carandini M. (2017). Focal cortical seizures start as standing waves and propagate respecting homotopic connectivity. Nat. Commun. 8:217. 10.1038/s41467-017-00159-6 28794407 PMC5550430

[B60] Schevon C. A. Weiss S. A. McKhann G. Jr. Goodman R. R. Yuste R. Emerson R. G.. (2012). Evidence of an inhibitory restraint of seizure activity in humans. Nat. Commun. 3:1060. 10.1038/ncomms2056 22968706 PMC3658011

[B61] Sessolo M. Marcon I. Bovetti S. Losi G. Cammarota M. Ratto G. M.. (2015). Parvalbumin-positive inhibitory interneurons oppose propagation but favor generation of focal epileptiform activity. J. Neurosci. 35, 9544–9557. 10.1523/JNEUROSCI.5117-14.2015 26134638 PMC6605139

[B62] Shiri Z. Manseau F. Lévesque M. Williams S. Avoli M. (2015). Interneuron activity leads to initiation of low-voltage fast-onset seizures. Ann. Neurol. 77, 541–546. 10.1002/ana.24342 25546300 PMC4880461

[B63] Smith E. H. Liou J. Davis T. S. Merricks E. M. Kellis S. S. Weiss S. A.. (2016). The ictal wavefront is the spatiotemporal source of discharges during spontaneous human seizures. Nat. Commun. 7:11098. 10.1038/ncomms11098 27020798 PMC4820627

[B64] Sorokin J. M. Davidson T. J. Frechette E. Abramian A. M. Deisseroth K. Huguenard J. R.. (2016). Bidirectional control of generalized epilepsy networks via rapid real-time switching of firing mode. Neuron 93, 194–210. 10.1016/j.neuron.2016.11.026 27989462 PMC5268077

[B65] Szabo G. G. Schneider C. J. Soltesz I. (2015). Resolution revolution: epilepsy dynamics at the microscale. Curr. Opin. Neurobiol. 31, 239–243. 10.1016/j.conb.2014.12.012 25596364 PMC4375062

[B67] Trevelyan A. J. Sussillo D. Watson B. O. Yuste R. (2006). Modular propagation of epileptiform activity: evidence for an inhibitory veto in neocortex. J. Neurosci. 26, 12447–12455. 10.1523/jneurosci.2787-06.2006 17135406 PMC6674895

[B66] Trevelyan A. J. Sussillo D. Yuste R. (2007). Feedforward inhibition contributes to the control of epileptiform propagation speed. J. Neurosci. 27, 3383–3387. 10.1523/jneurosci.0145-07.2007 17392454 PMC6672122

[B68] Truccolo W. Donoghue J. A. Hochberg L. R. Eskandar E. N. Madsen J. R. Anderson W. S.. (2011). Single-neuron dynamics in human focal epilepsy. Nat. Neurosci. 14, 635–641. 10.1038/nn.2782 21441925 PMC3134302

[B69] Tsien R. Y. (1981). A non-disruptive technique for loading calcium buffers and indicators into cells. Nature 290, 527–528. 10.1038/290527a0 7219539

[B91] van den Maagdenberg A. M. J. M Pietrobon D. Pizzorusso T. Kaja S. Broos L. A. M. Cesetti T.. (2004). A *Cacna1a* knockin migraine mouse model with increased susceptibility to cortical spreading depression. Neuron 41, 701–710. 10.1016/S0896-6273(04)00085-6 15003170

[B70] Viventi J. Kim D. H. Vigeland L. Frechette E. S. Blanco J. A. Kim Y. S.. (2011). Flexible, foldable, actively multiplexed, high-density electrode array for mapping brain activity *in vivo*. Nat. Neurosci. 14, 1599–1605. 10.1038/nn.2973 22081157 PMC3235709

[B71] Wenzel M. Hamm J. P. Peterka D. S. Yuste R. (2017). Reliable and elastic propagation of cortical seizures *in vivo*. Cell Rep. 19, 2681–2693. 10.1016/j.celrep.2017.05.090 28658617 PMC5551439

[B72] Wiebe S. Jette N. (2012). Pharmacoresistance and the role of surgery in difficult to treat epilepsy. Nat. Rev. Neurol. 8, 669–677. 10.1038/nrneurol.2012.181 22964510

[B73] Wykes R. C. Heeroma J. H. Mantoan L. Zheng K. MacDonald D. C. Deisseroth K.. (2012). Optogenetic and potassium channel gene therapy in a rodent model of focal neocortical epilepsy. Sci. Transl. Med. 4:161ra152. 10.1126/scitranslmed.3004190 23147003 PMC3605784

[B74] Xiao D. Vanni M. P. Mitelut C. C. Chan A. W. Ledue J. M. Xie Y.. (2017). Mapping cortical mesoscopic networks of single spiking cortical or sub-cortical neurons. Elife 6:e19976. 10.7554/elife.19976 28160463 PMC5328594

[B76] Yang W. Miller J. E. Carrillo-Reid L. Pnevmatikakis E. Paninski L. Yuste R.. (2016). Simultaneous multi-plane imaging of neural circuits. Neuron 89, 269–284. 10.1016/j.neuron.2015.12.012 26774159 PMC4724224

[B75] Yang W. Yuste R. (2017). *In vivo* imaging of neural activity. Nat. Methods 14, 349–359. 10.1038/nmeth.4230 28362436 PMC5903578

[B77] Yekhlef L. Breschi G. L. Lagostena L. Russo G. Taverna S. (2015). Selective activation of parvalbumin- or somatostatin-expressing interneurons triggers epileptic seizurelike activity in mouse medial entorhinal cortex. J. Neurophysiol. 113, 1616–1630. 10.1152/jn.00841.2014 25505119

[B78] Yu F. H. Mantegazza M. Westenbroek R. E. Robbins C. A. Kalume F. Burton K. A.. (2006). Reduced sodium current in GABAergic interneurons in a mouse model of severe myoclonic epilepsy in infancy. Nat. Neurosci. 9, 1142–1149. 10.1038/nn1754 16921370

